# Fibrosis-4 and Aspartate Aminotransferase-to-Platelet Ratio Index as Predictors of Relapse in Autoimmune Hepatitis: A Retrospective Single-Center Study

**DOI:** 10.5152/tjg.2026.26177

**Published:** 2026-06-25

**Authors:** İlyas Ethem Şenocak, Yunus Günegül, Yunus Emre Demiral, Talha Ercan, Ahmet Tarık Eminler

**Affiliations:** Department of Gastroenterology, Sakarya University Faculty of Medicine, Sakarya, Türkiye

**Keywords:** APRI, autoimmune hepatitis, FIB-4, noninvasive fibrosis score, relapse

## Abstract

**Background/Aims:**

Autoimmune hepatitis (AIH) is associated with a high rate of relapse following treatment withdrawal. Identifying noninvasive markers that predict disease recurrence is crucial for long-term management. This study evaluated the prognostic performance of the Fibrosis-4 (FIB-4) Index and the Aspartate Aminotransferase-to-Platelet Ratio Index (APRI) in predicting post-treatment relapse in AIH.

**Materials and Methods:**

This retrospective, single-center cohort study included 65 patients with AIH between January 2010 and October 2025 who achieved sustained normalization of transaminase under corticosteroid-based therapy and were followed up for at least 12 months. Baseline demographic, laboratory, and noninvasive fibrosis parameters were analyzed. Relapse was defined as alanine aminotransferase or aspartate aminotransferase elevation ≥2 times the upper limit of normal and/or serum immunoglobulin G (IgG) above the upper limit, occurring after complete discontinuation of immunosuppressive therapy. Receiver operating characteristic (ROC) curve analysis and logistic regression were performed.

**Results:**

Relapse occurred in 26 patients (40.0%). Patients with relapse had higher baseline serum IgG (*P* < .001), lower platelet counts (*P* = .004), and higher APRI and FIB-4 scores (*P* < .001 for both). ROC analysis identified optimal cutoff values of APRI >1.54 (area under the curve (AUC) = 0.81) and FIB-4 > 3.84 (AUC = 0.80). In multivariate analysis, baseline IgG (odds ratio (OR) = 1.09; 95% CI = 1.00-1.18; *P* = .049) and FIB-4 > 3.84 (OR = 6.68; 95% CI = 2.02-22.10; *P* = .002) remained independent predictors.

**Conclusion:**

Baseline FIB-4 and serum IgG independently predict relapse following discontinuation of immunosuppressive therapy in AIH and may support individualized follow-up strategies.

**Main Points**

- Baseline serum immunoglobulin G levels at diagnosis and baseline Fibrosis-4 (FIB-4) scores were identified as independent predictors of relapse.
- A baseline FIB-4 score above the optimal cutoff value of 3.84 is associated with a higher probability of disease recurrence following treatment withdrawal.
- The negative predictive values of FIB-4 and Aminotransferase-to-Platelet Ratio Index help identify patients at low risk of relapse and may assist in clinical decision-making regarding treatment continuation vs. withdrawal.

## Introduction

Autoimmune hepatitis (AIH) is a chronic inflammatory liver disease characterized by elevated transaminase levels, the presence of circulating autoantibodies, and increased serum immunoglobulin G (IgG) concentrations.^1^^,^^2^ The primary therapeutic goal in AIH is to achieve complete biochemical remission and prevent progression to cirrhosis and hepatocellular carcinoma. Despite successful treatment, AIH is associated with a substantial risk of relapse, with reported rates ranging from 30% to 50% after withdrawal of immunosuppressive therapy or during maintenance treatment.^3^^,^^4^ Identifying patients at increased risk of relapse is essential for optimizing treatment duration and improving monitoring strategies.^5^ Although liver biopsy remains the reference standard for assessing hepatic inflammation and fibrosis, its invasive nature, potential sampling variability, and limited patient acceptance restrict its routine use in longitudinal disease monitoring.^6^ Consequently, there is a growing need for reliable, noninvasive, and readily accessible biomarkers that can predict disease recurrence and assist in clinical decision-making.

Increasing attention has focused on immune and serum-based biomarkers for relapse prediction in AIH, as well as clinical markers for risk stratification and outcome assessment in autoimmune and related liver diseases.^7^^,^^8^ In parallel, fibrosis-based indices such as Aminotransferase-to-Platelet Ratio Index (APRI) and Fibrosis-4 (FIB-4)—originally developed for viral hepatitis and metabolic dysfunction–associated steatotic liver disease (MASLD)—have been explored in AIH because of their ability to reflect both structural liver injury and inflammatory activity.^9^^,^^10^ However, their predictive value for relapse in AIH remains limited and inconsistent.

Despite established immunosuppressive regimens, approximately 10%-20% of patients with AIH fail to achieve or maintain complete biochemical response or are intolerant to first-line therapy, constituting a heterogeneous subgroup defined as difficult-to-treat AIH.^11^^,^^12^ This clinically distinct population poses particular challenges in predicting long-term outcomes, including relapse.

Therefore, the aim of the present study was to evaluate the performance of baseline APRI and FIB-4 scores in predicting relapse in patients with AIH and to determine clinically relevant cutoff values that may assist clinicians in identifying patients at higher risk of recurrence.

## Materials and Methods

### Study Design and Population

This retrospective cohort study included patients diagnosed with AIH at Department of Gastroenterology, Sakarya University Faculty of Medicine, Sakarya, Türkiye between January 2010 and October 2025.

Patients diagnosed with AIH according to the simplified criteria of the International Autoimmune Hepatitis Group (IAIHG) who achieved sustained normalization of transaminase levels under corticosteroid-based therapy and were followed up for at least 12 months were included in the study.

Patients with other chronic liver diseases, including viral hepatitis, alcoholic liver disease, MASLD, biliary disorders, Wilson’s disease, alpha-1 antitrypsin deficiency, hemochromatosis, and drug-induced liver injury, as well as those aged <18 years and pregnant women, were excluded. Drug-induced AIH was strictly excluded using Roussel Uclaf Causality Assessment Method-based assessment and a detailed medication history to ensure the idiopathic nature of the cohort. Occurrence of relapse after treatment withdrawal further supported the diagnosis of idiopathic AIH.^13^

Patient selection is summarized in the study flow. A total of 96 patients diagnosed with AIH between January 2010 and October 2025 were retrospectively evaluated. Seventeen patients were excluded due to missing data, and 11 were excluded for difficult-to-treat AIH, defined as failure to achieve sustained transaminase normalization within 12 months or insufficient follow-up. In addition, 3 patients with biochemical reactivation during ongoing immunosuppressive therapy were excluded from the primary relapse analysis. Consequently, 65 patients were included in the final analysis.

Sustained transaminase normalization was defined as the persistent normalization of both alanine aminotransferase (ALT) and aspartate aminotransferase (AST) levels (≤1× upper limit of normal (ULN)) on at least 2 consecutive measurements during follow-up. For all analyses, the ULN for aminotransferases was standardized as 35 U/L, in accordance with the institutional laboratory reference range.

In this study, sustained transaminase normalization, rather than complete biochemical remission, was used as the inclusion criterion. The rationale for prioritizing aminotransferase normalization over IgG normalization is addressed in the “Discussion” section.^12^^,^^14^

Relapse was defined as biochemical reactivation occurring after complete discontinuation of immunosuppressive therapy, characterized by ALT or AST ≥2 × ULN and/or serum IgG above the ULN. This threshold was selected in accordance with previously established criteria to facilitate early detection of postdiscontinuation relapse.^15^^,^^16^

Three patients with biochemical reactivation during ongoing immunosuppressive therapy (ALT or AST ≥3 × ULN) were excluded from the primary relapse analysis to maintain a homogeneous study population focused on postdiscontinuation relapse as the primary outcome.^17^

The primary endpoint of the study was the cumulative incidence of relapse during the follow-up period after achieving sustained transaminase normalization. The secondary endpoint was the identification and characterization of demographic, clinical, and laboratory parameters associated with or potentially contributing to the development of relapse.

### Treatment and Follow-up Protocol

Treatment followed international clinical practice guidelines.^6^^,^^17^ Initial therapy consisted of prednisolone (0.5-1 mg/kg/day) with or without azathioprine (50-100 mg/day). Following biochemical remission, steroids were tapered to a maintenance dose of 5-7.5 mg/day. Treatment modifications were based on clinical and biochemical response. Patients were monitored every 3 to 6 months with clinical and laboratory assessments.

To ensure accurate relapse diagnosis, patients with biochemical reactivation were systematically evaluated for alternative causes, including viral infections, drug-induced liver injury, and metabolic factors. Relapse was confirmed by exclusion of these causes and a favorable response to reinitiation or escalation of immunosuppressive therapy.

### Data Collection

Medical records of patients diagnosed with AIH were reviewed retrospectively. Noninvasive fibrosis indices (APRI and FIB-4) were calculated exclusively using laboratory parameters (AST, ALT, platelet count, and age) obtained at the time of initial AIH diagnosis (baseline), before the initiation of any immunosuppressive therapy. No subsequent recalculation was performed during follow-up, treatment tapering, or at the time of relapse, as the aim of this study was to evaluate the prognostic value of baseline fibrosis indices for predicting future relapse, rather than their utility as dynamic monitoring tools. By systematically reviewing medical records, the following demographic and clinical variables were extracted: patient characteristics, date of AIH diagnosis, total follow-up duration, serum ALT, AST, alkaline phosphatase, gamma-glutamyl transferase, total bilirubin, albumin, international normalized ratio, IgG, platelet count, autoantibody profiles (ANA, SMA, AMA, LKM-1, and SLA/LP), and detailed information regarding immunosuppressive treatment. LKM-1 and SLA/LP were not systematically tested in all patients because of the retrospective study design and local clinical protocols, in which these assays were primarily performed in ANA/SMA-negative cases. In addition, histological data, including the Histological Activity Index (HAI) and fibrosis stage, were recorded for patients who underwent liver biopsy at diagnosis.

Noninvasive fibrosis indices were calculated using the following validated formulas:



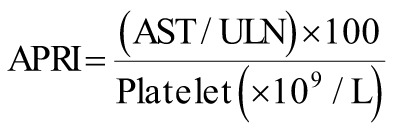







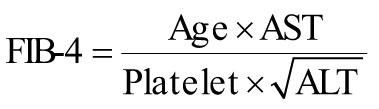



### Statistical Analysis

Statistical analyses were performed using IBM SPSS Statistics for Windows, version 26.0 (IBM SPSS Corp.; Armonk, NY, USA). Descriptive statistics are presented as mean ± SD for normally distributed continuous variables and as median (minimum–maximum) for non-normally distributed variables. Categorical variables are expressed as numbers and percentages. The normality of data was assessed using the Shapiro–Wilk test. Comparisons between groups were performed using the Student’s *t*-test for normally distributed data and the Mann–Whitney *U*-test for non-normal data. Categorical data were compared using the chi-square test or Fisher’s exact test where appropriate. Receiver operating characteristic (ROC) curve analysis was used to determine the diagnostic performance and optimal cutoff values of noninvasive scores. Logistic regression analysis was performed to identify independent predictors of relapse. A *P* value of less than .05 was considered statistically significant. Baseline serum IgG was entered into the logistic regression model as a continuous variable, and the corresponding odds ratio (OR) was interpreted per 1 g/L increase in baseline IgG concentration.

**Ethics Approval**

This study was approved by the Ethics Committee of Sakarya University Faculty of Medicine (approval number 43012747-050.04-523212-499 ; date: October 10, 2025). The study was conducted in accordance with the principles of the Declaration of Helsinki (2013 revision). Written informed consent was waived due to the retrospective design of the study. During the preparation of this manuscript, the authors used large language model–based generative artificial intelligence tools solely for language editing, grammar improvement, and improving readability. The authors reviewed, edited, and approved all generated output and take full responsibility for the content of the manuscript. 

## Results

The final study cohort included 65 patients with AIH. Baseline demographic and clinical characteristics are summarized in Table 1. The mean age was 54.4 ± 14.8 years, 49 patients (75.4%) were female, and the median follow-up duration was 84 months (range = 24-144 months). ANA and/or SMA positivity was present in 60 patients (92.3%), consistent with a predominantly type 1 AIH cohort.

Initial treatment consisted of steroids plus azathioprine in 66.2% of patients (n = 43) and steroid monotherapy in 12.3% (n = 8). The remaining 21.5% (n = 14) received alternative regimens, mainly budesonide with azathioprine or mycophenolate mofetil, with 2 patients receiving cyclosporine-based therapy (Table 1). These regimens were initiated due to comorbidities or contraindications to prednisolone, and all patients achieved biochemical remission within 12 months.

After treatment discontinuation, 26 patients (40.0%) experienced relapse, while 39 (60.0%) maintained sustained biochemical response. All relapse events occurred exclusively after complete cessation of immunosuppressive therapy, with no reactivation during tapering or treatment transition. At diagnosis, 21.5% of patients had biochemically severe AIH and 43.1% exhibited high histological activity (HAI ≥12).

Histological assessment was available in 51 patients (78.5%) at diagnosis. The relapse group showed significantly higher necroinflammatory activity (HAI: 13.4 ± 3.2 vs. 11.2 ± 2.8; *P* = .012) and fibrosis stage (Ishak: 3.2 ± 1.1 vs. 2.1 ± 0.9; *P* = .004). These findings were consistent with baseline FIB-4–defined high-risk fibrosis categories.

The median duration of therapy before treatment discontinuation was 54 months. As shown in Table 2, comparative analysis revealed that biochemical parameters at the time of treatment discontinuation—including ALT, AST, and IgG levels—were nearly identical and within normal limits for both the relapse and sustained response groups (*P* > .05 for all). This indicates that all patients achieved complete biochemical remission and maintained this state for a sufficient duration before stopping therapy.

Age, sex, and initial treatment regimens were comparable between groups. Patients who relapsed had higher baseline serum IgG levels (26.2 ± 8.4 vs. 20.5 ± 4.9 g/L; *P* < .001) and lower platelet counts (179.4 ± 84.1 vs. 234.5 ± 67.8 × 10^9^/L; *P* = .004). Concomitant autoimmune diseases and time to biochemical remission did not differ significantly between groups (*P* > .05). LKM-1, SLA/LP, and serum globulin levels were not systematically available in all patientsand were excluded from comparative analysis. Baseline noninvasive fibrosis scores were significantly higher in patients who relapsed (APRI: 4.5 (range = 1.5-9.1) vs. 0.9 (range = 0.6-1.5); FIB-4: 5.0 (range = 3.3-9.2) vs. 2.2 (range = 1.2-3.4); *P* < .001 for both).

**Figure 1.**
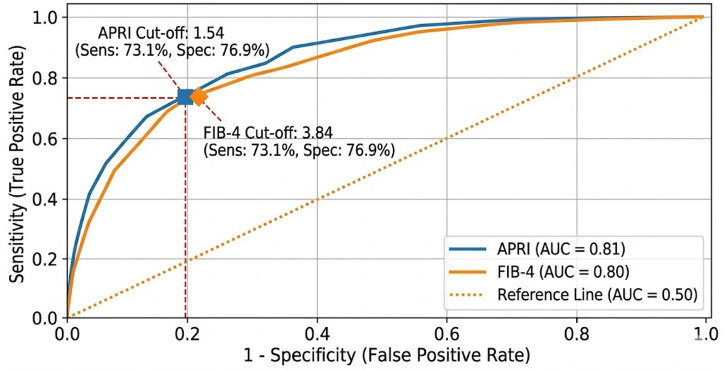
Receiver operating characteristic curves of baseline noninvasive indices for predicting relapse in patients with autoimmune hepatitis. The Aspartate Aminotransferase-to-Platelet Ratio Index (APRI) at diagnosis yielded an area under the curve (AUC) of 0.81 (cutoff: 1.54; sensitivity: 73.1%; specificity: 76.9%), and Fibrosis-4 (FIB-4) at diagnosis yielded an AUC of 0.80 (cutoff: 3.84; sensitivity: 73.1%; specificity: 76.9%). Both indices significantly predicted relapse (*P* < .001). The diagonal reference line indicates AUC = 0.50.

ROC curve analysis demonstrated strong discriminative performance for predicting relapse, with an area under the curve (AUC) of 0.81 for APRI and 0.80 for FIB-4 (Figure 1). According to the Youden index, the optimal cutoff values for predicting relapse were 1.54 for APRI (sensitivity 73.1%, specificity 76.9%) and 3.84 for FIB-4 (sensitivity 73.1%, specificity 76.9%). Both indices demonstrated reliable predictive capacity, with a negative predictive value of 81.1% for both markers (Table 3).

In the univariable logistic regression analysis, APRI >1.54 (OR = 9.05, *P* < .001), FIB-4 > 3.84 (OR = 9.05, *P* < .001), and baseline serum IgG level, analyzed as a continuous variable per 1 g/L increase (OR = 1.13, *P* = .005), were significant predictors of relapse risk. A low platelet count (<150 × 10^9^/L) demonstrated a trend toward an association with relapse risk but did not reach statistical significance (*P* = .078).

In the multivariable logistic regression model, FIB-4 > 3.84 (OR = 6.68, *P* = .002) and baseline serum IgG level per 1 g/L increase (OR = 1.09, *P* = .049) remained independently associated with relapse. Although APRI > 1.54 was a strong predictor in the univariable analysis, it was not included in the final multivariable model because of collinearity with FIB-4, given their identical OR values and overlapping performance. The results of the regression analyses are summarized in Table 4.

## Discussion

AIH is characterized by a relapsing course, with reported relapse rates ranging between 30% and 50%. In the present study, the relapse rate was 40.0%, consistent with previous reports. Identifying predictors of relapse is important because recurrent disease activity is associated with accelerated hepatic fibrosis, progression to cirrhosis, and increased liver-related morbidity. Therefore, achieving and maintaining complete biochemical remission remains the primary goal of treatment to prevent long-term complications.^6^

Previous studies have highlighted the high risk of relapse after withdrawal of immunosuppressive therapy in AIH. In a large multicenter Dutch cohort, van Gerven et al reported that relapse or loss of remission occurred in most patients after treatment withdrawal, with 59% requiring retreatment within 1 year and 81% within 3 years.^18^ In contrast, Hartl et al demonstrated that more stringent patient selection, including sustained complete biochemical remission while receiving immunosuppressive monotherapy for at least 2 years, may substantially improve the likelihood of maintaining remission after treatment withdrawal. These findings suggest that treatment withdrawal in AIH should be approached cautiously and that reliable predictors are needed to identify patients at increased risk of relapse.^19^In this context, the present study adds to the existing literature by demonstrating that baseline FIB-4 and serum IgG levels, assessed at diagnosis, may provide additional prognostic information for predicting relapse after withdrawal of immunosuppressive therapy.

Elevated baseline serum IgG levels and a high FIB-4 score were identified as independent predictors of relapse in the present cohort. Although thrombocytopenia was associated with relapse in univariate analysis, this association did not remain significant in multivariate analysis (OR = 2.91; 95% CI = 0.89-9.54; *P* = .078), likely reflecting collinearity with FIB-4. Elevated IgG may reflect immunological disease activity at presentation, whereas lower platelet counts may indicate greater fibrosis-related disease burden.^12^^,^^20^ In multivariate analysis, baseline IgG and FIB-4 > 3.84 remained the strongest independent predictors.

In the cohort, baseline serum IgG at diagnosis was significantly higher in patients who subsequently relapsed. This finding is not inconsistent with the inclusion criterion of sustained transaminase normalization, as these parameters reflect different temporal and biological aspects of the disease. Baseline IgG reflects the immunological activity at presentation, whereas transaminase normalization indicates treatment response.^21^ Recent evidence suggests that the prognostic value of IgG normalization during therapy is limited.^11^^,^^12^ Accordingly, elevated baseline IgG at diagnosis likely represents a more aggressive disease phenotype with an increased risk of relapse after treatment withdrawal.^22^ This supports the concept that baseline immunological burden provides independent prognostic information.^11^

The prognostic role of IgG in AIH remains debated. The IAIHG retrospective registry identified failure to achieve complete biochemical response as a predictor of adverse outcomes but did not distinguish between aminotransferase and IgG normalization.^23^ More recent data suggest that aminotransferase normalization may be the primary determinant during treatment, while the significance of persistent IgG elevation remains unclear.^12^^,^^24^

Unlike prior studies assessing on-treatment IgG, baseline IgG at diagnosis was evaluated as a prognostic marker. From this perspective, elevated baseline IgG likely reflects a stable marker of disease aggressiveness and retains predictive value for relapse regardless of subsequent IgG normalization. This interpretation integrates the findings within the current debate and underscores the need for prospective studies evaluating both baseline and treatment-related IgG dynamics.

Although APRI and FIB-4 were originally developed for viral hepatitis and MASLD, their utility in AIH is increasingly recognized. A meta-analysis of over 2000 patients demonstrated moderate diagnostic performance for fibrosis assessment, with FIB-4 showing slightly superior accuracy, particularly for cirrhosis.^20^ More recent studies indicate that APRI may reliably exclude advanced fibrosis and cirrhosis during treatment.^25^ Additionally, cirrhosis at diagnosis has been identified as a strong predictor of adverse outcomes, underscoring the importance of accurate baseline fibrosis assessment using accessible noninvasive indices.^26^ Previous reports also suggest superior performance of FIB-4 over APRI for cirrhosis detection.^27^ Extending these findings, the results indicate that both indices are higher in patients who develop relapse.

Noninvasive fibrosis indices such as FIB-4 are influenced by transaminase levels and may reflect inflammatory activity rather than true fibrosis, particularly in acute settings.^28^ Similarly, high necroinflammatory activity may lead to overestimation of structural liver damage.^29^ Accordingly, elevated baseline FIB-4 and APRI values in the cohort likely reflect disease aggressiveness and inflammatory burden, which may contribute to increased relapse risk.

Specifically, the results demonstrated that an FIB-4 score >3.84 and an APRI score >1.54 at baseline are robust predictors of disease recurrence. This finding adds a novel dimension to the existing literature, as previous AIH studies on APRI and FIB-4 have predominantly focused on cross-sectional fibrosis estimation and on the exclusion of advanced fibrosis during treatment, whereas relatively few have evaluated their longitudinal prognostic value for predicting post-treatment relapse.^20^^,^^25^ These findings suggest that baseline fibrosis-related indices reflect not only structural damage but also a pro-inflammatory milieu that predisposes patients to relapse after achieving biochemical remission.^30^^,^^31^

The limitations and challenges of histological assessment in AIH underscore the value of noninvasive prognostic markers. Although liver biopsy remains the diagnostic reference standard, it is invasive, carries procedural risks, and is unsuitable for serial monitoring.^6^^,^^17^ Histological assessment is further limited by intercriteria variability, sampling error, and observer variability, as well as challenges in distinguishing AIH from other causes of hepatitis.^12^^,^^32^ In the cohort, although most patients underwent biopsy at diagnosis, samples were occasionally inadequate for fibrosis staging and repeat biopsy was rarely performed.

In this context, simple, reproducible, and low-cost serum-based indices such as APRI and FIB-4 provide a valuable complementary approach, offering noninvasive, repeatable, and observer-independent assessment, with independent prognostic value for post-treatment relapse risk. These indices should be considered complementary to, rather than replacements for, histology, particularly in real-world settings where repeated biopsy is impractical.

This study has several limitations. Its retrospective, single-center design and relatively small sample size may limit generalizability and statistical power. Some laboratory and serological parameters, including serum globulin levels and LKM-1/SLA/LP antibodies, were not systematically assessed, resulting in a cohort predominantly composed of patients with type 1 AIH. Although most patients underwent liver biopsy, histological data were unavailable for all patients and some samples were insufficient for fibrosis staging; therefore, validated noninvasive criteria were used to standardize baseline fibrosis risk assessment. APRI and FIB-4 were calculated at diagnosis, when elevated transaminase levels may overestimate fibrosis stage; however, the findings suggest that these indices may also reflect overall disease activity and biological aggressiveness rather than static fibrosis alone. Relapse was defined biochemically and was not histologically confirmed in all cases, reflecting real-world practice. Patients with difficult-to-treat AIH were excluded to preserve cohort homogeneity; therefore, the proposed cutoffs (APRI > 1.54 and FIB-4 > 3.84) should not be extrapolated to this subgroup.^11^ Finally, although APRI and FIB-4 are well validated in viral hepatitis, the findings support their prognostic relevance in AIH, particularly for relapse prediction.

## Conclusion

Baseline FIB-4 and serum IgG independently predict relapse following discontinuation of immunosuppressive therapy in AIH. Elevated baseline IgG levels and FIB-4 >3.84 were independently associated with relapse risk, while thrombocytopenia—although significant in univariate analysis—did not remain significant in multivariate analysis. These findings support the use of simple laboratory-based indices for improved risk stratification and individualized follow-up. Prospective multicenter studies are needed to validate these results and clarify the role of serial monitoring.

## Figures and Tables

**Table 1.** Demographic and Clinical Characteristics of the Study Population (n = 65)

Variable	Total (n = 65)
Demographic data	
Age, years (mean ± SD)	54.4 ± 14.8
Female sex, n (%)	49 (75.4)
Clinical and disease severity	
Follow-up duration, months (median, range)	84 (24-144)
Biochemically severe AIH*, n (%)	14 (21.5)
High histological activity**, n (%)	28 (43.1)
True relapse (postdiscontinuation), n (%)	26 (40.0)
Sustained biochemical response, n (%)	39 (60.0)
Laboratory parameters at diagnosis (pretreatment)	
ALT, U/L (mean ± SD)	238.6 ± 312.4
AST, U/L (mean ± SD)	215.8 ± 265.1
Platelet count, × 10^9^/L (mean ± SD)	212.4 ± 78.6
Serum IgG, g/L (mean ± SD)	22.8 ± 6.9
Noninvasive fibrosis scores (at diagnosis)	
APRI, median (range)	1.6 (0.6-9.1)
FIB-4, median (range)	2.8 (1.2-9.2)
Initial treatment regimen	
Steroid + azathioprine, n (%)	43 (66.2)
Steroid monotherapy, n (%)	8 (12.3)
Others/combinations, n (%)***	14 (21.5)

AIH, autoimmune hepatitis; ALT, alanine aminotransferase; APRI, Aspartate Aminotransferase-to-Platelet Ratio Index; AST, aspartate aminotransferase; FIB-4, Fibrosis-4 Index; HAI, Histological Activity Index; IgG, immunoglobulin G; ULN, upper limit of normal.

*Defined as transaminases >10 times the upper limit of normal at diagnosis.

**Defined as HAI score ≥12 and/or presence of bridging or submassive necrosis (available for n = 51).

***Alternative regimens included budesonide + azathioprine (n = 6), mycophenolate mofetil + steroid (n = 4), mycophenolate mofetil monotherapy (n = 2), and cyclosporine-based regimens (n = 2).

**Table 2.** Comparison of Baseline Clinical and Laboratory Characteristics Between Patients With and Without Relapse (n = 65)

Variable	Relapse (+) (n = 26)	Relapse (−) (n = 39)	*P*
Demographic characteristics			
Age, years (mean ± SD)	56.8 ± 14.5	52.8 ± 15.3	.284
Female sex, n (%)	18 (69.2)	31 (79.5)	.518
Baseline clinical status			
High risk for advanced fibrosis*, n (%)	9 (34.6)	3 (7.7)	.008
Laboratory parameters at diagnosis (pretreatment)			
ALT, U/L (mean ± SD)	321.4 ± 325.2	183.4 ± 294.6	.082
AST, U/L (mean ± SD)	315.8 ± 308.1	149.2 ± 210.4	.012
Platelet count, × 10^9^/L (mean ± SD)	179.4 ± 84.1	234.5 ± 67.8	.004
Serum IgG, g/L (mean ± SD)	26.2 ± 8.4	20.5 ± 4.9	<.001
Histological findings			
Histological Activity Index	13.4 ± 3.2	11.2 ± 2.8	.012
Fibrosis stage (Ishak 0-6)	3.2 ± 1.1	2.1 ± 0.9	.004
Advanced fibrosis (FIB-4 > 3.25)	11 (42.3)	6 (15.4)	.017
Initial treatment regimen			
Steroid + azathioprine, n (%)	20 (76.9)	23 (59.0)	.142
Steroid monotherapy, n (%)	3 (11.5)	5 (12.8)	.874
Noninvasive scores at diagnosis			
APRI, median (range)	4.5 (1.5-9.1)	0.9 (0.6-1.5)	<.001
FIB-4, median (range)	5.0 (3.3-9.2)	2.2 (1.2-3.4)	<.001
Characteristics at treatment discontinuation			
Duration of therapy, months (median)	52.5	56.0	.412
ALT at discontinuation, U/L (mean ± SD)	19.4 ± 8.2	18.1 ± 7.5	.524
AST at discontinuation, U/L (mean ± SD)	21.2 ± 9.1	20.4 ± 8.4	.718
IgG at discontinuation, g/L (mean ± SD)	14.8 ± 3.2	13.5 ± 2.8	.084

ALT, AST, platelet count, and IgG are reported as mean ± SD due to approximately normal distribution; APRI and FIB-4 are presented as median (min–max).

ALT, alanine aminotransferase; APRI, Aspartate Aminotransferase-to-Platelet Ratio Index; AST, aspartate aminotransferase; FIB-4, Fibrosis-4 index; HAI, Histological Activity Index; IgG, immunoglobulin G; Ishak, Ishak fibrosis stage.

*Defined as FIB-4 > 3.25 and platelet count < 150 × 10^9^/L at the time of diagnosis. The 3.25 threshold for advanced fibrosis was applied as the established cutoff in the general fibrosis literature (Sterling et al, Hepatology 2006), distinct from the FIB-4 cutoff of 3.84 derived from the own ROC analysis for predicting relapse.

**Table 3.** Diagnostic Performance of APRI and FIB-4 Based on ROC Curve Analysis

Parameter	AUC	Cutoff	Sensitivity (%)	Specificity (%)	PPV (%)	NPV (%)	*P*
APRI	0.81	1.54	73.1	76.9	67.9	81.1	<.001
FIB-4	0.80	3.84	73.1	76.9	67.9	81.1	<.001

APRI, Aspartate Aminotransferase-to-Platelet Ratio Index; AUC, area under the curve; FIB-4, Fibrosis-4 Index; NPV, negative predictive value; PPV, positive predictive value; ROC, receiver operating characteristic.

**Table 4.** Univariate and Multivariate Logistic Regression Analysis for Predictors of Relapse

Variable	Univariate OR (95% CI)	*P*	Multivariate OR (95% CI)	*P*
APRI > 1.54	9.05 (2.89-28.37)	<.001	—	—
FIB-4 > 3.84	9.05 (2.89-28.37)	<.001	6.68 (2.02-22.10)	.002
Serum IgG (baseline)	1.13 (1.04-1.23)	.005	1.09 (1.00-1.18)	.049
Low platelet count (<150 × 10^9^/L)	2.91 (0.89-9.54)	.078	—	—

Variables with *P* < .10 in univariate analysis were included in the multivariate logistic regression model.

For serum IgG, odds ratios represent the change in relapse risk per 1 g/L increase in baseline IgG concentration.

APRI, Aspartate Aminotransferase-to-Platelet Ratio Index; FIB-4, Fibrosis-4 Index; IgG, immunoglobulin G, OR, odds ratio.

## Data Availability

The data that support the findings of this study are available on request from the corresponding author.
